# 8-Methoxybicolosin C from *Lespedeza bicolor* Attenuates Inflammation and Oxidative Stress via Nrf2/HO-1 and NF-κB/MAPK Pathways in Lipopolysaccharide-Induced Mouse Kupffer Cells

**DOI:** 10.4014/jmb.2503.03013

**Published:** 2025-08-18

**Authors:** Young-Chang Cho, Lulu Yao, Da Young Lee, Xiangying Li, Guijae Yoo, Sang Yoon Choi, Namki Cho, Su-Jin Park, Somy Yoon, Jae Sung Lim

**Affiliations:** 1 College of Pharmacy and Research Institute of Pharmaceutical Sciences, Chonnam National University, Gwangju 61186, Republic of Korea; 2 R&D Center, CUOME BIO Co., Ltd., Hwasun-gun, Jeollanam-do 58141, Republic of Korea; 3 Korea Food Research Institute, Wanju-Gun 55365, Jeollabuk-do, Republic of Korea; 4 Functional Biomaterial Research Center, Korea Research Institute of Bioscience and Biotechnology, Jeongeup-si 56212, Republic of Korea

**Keywords:** 8-Methoxybicolosin C, antioxidant, anti-inflammation, Nrf2/HO-1 pathway, NF-κB, MAPKs

## Abstract

*Lespedeza bicolor* (*L. bicolor*) is known for its anti-inflammatory, antioxidant, and anticancer properties, making it a common choice in traditional medicine practices. Researchers in several recent studies have focused on isolating individual phytochemicals from this plant through chromatography analysis to explore their therapeutic potential. In our previous work, we identified 8-methoxybicolosin C (8-MC) as a novel flavonoid derivative, isolated and purified from the roots of *L. bicolor*, which exhibited inhibitory effects on cell proliferation. In this study, we further investigated the biological activities of 8-MC by examining its antioxidant and anti-inflammatory effects in LPS-induced mouse Kupffer cells. The results showed that 8-MC suppresses the expression of inflammation-related mediators, including inducible nitric oxide synthase (iNOS), nitric oxide (NO), and pro-inflammatory cytokines such as tumor necrosis factor (TNF)-α, interleukin (IL)-6, and IL-1β, in a dose-dependent manner. Additionally, 8-MC improves the GSH/GSSG balance by increasing glutathione (GSH) levels and decreasing oxidized glutathione (GSSG) levels. Interestingly, 8-MC was found to bind Keap1, preventing roteasomal degradation, and promoting the nuclear translocation of nuclear factor erythroid 2-related factor 2 (Nrf2), thereby increasing the expression of antioxidant-related proteins such as heme oxygenase-1 (HO-1). Moreover, 8-MC suppressed the activation of inflammatory signaling pathways, including c-Jun N-terminal kinases (JNKs) and p38 mitogen-activated protein kinases (MAPKs), while also inhibiting the nuclear translocation of nuclear factor kappa B (NF-κB), effectively reducing inflammatory responses. These findings collectively demonstrated that 8-MC possesses potent anti-inflammatory and antioxidant activities through the regulation of NF-κB, MAPK, and Nrf2/HO-1 signaling pathways. Consequently, 8-MC shows potential as a valuable therapeutic agent for managing various inflammatory disorders.

## Introduction

Immune cells, such as macrophages, dendritic cells, and natural killer cells, play an essential role in protecting various body tissues, often triggering inflammation [1, 2]. Inflammation serves as the body’s initial physiological defense mechanism in response to injury, infection, and oxidative stress [3, 4]. In general, acute inflammation, which occurs in response to injury or infection, temporarily recruits immune cells and increases the production of inflammatory substances. This activation leads to cellular and molecular interactions that play a critical role in restoring tissue homeostasis and effectively controlling acute inflammation [4, 5]. However, if acute inflammation remains uncontrolled, it can progress into a chronic state, potentially driving the development of chronic inflammatory conditions such as liver fibrosis, diabetes, non-alcoholic steatohepatitis (NASH), cancer, and aging [6-9]. Consequently, regulating inflammation in immune cells is crucial for mitigating the risk of sustained tissue damage and associated diseases. Macrophages express various toll-like receptors (TLRs) on their cell membranes, which detect pathogen patterns [10]. Notably, TLR4 holds a specific role in recognizing lipopolysaccharide (LPS), a key component of gram-negative bacterial cell walls [11]. Upon LPS activation, macrophages initiate intracellular signaling pathways, encompassing nuclear factor kappa B (NF-κB) and mitogen-activated protein kinase (MAPK) pathways, resulting in the synthesis of nitric oxide (NO) and pro-inflammatory cytokines [12-14].

Oxidative stress, alongside inflammation, is closely linked to the development of chronic inflammatory diseases [15]. Free radicals, produced as part of the host defense response, accumulate and intensify oxidative stress by interacting with cellular components, leading to cell damage and inflammation [16, 17]. Consequently, reducing oxidative stress is pivotal for ameliorating inflammation. Nuclear factor erythroid 2-related factor 2 (Nrf2), a well-known transcription factor, is integral to preserving cellular homeostasis and providing protection against oxidative damage [18, 19]. Upon activation, Nrf2 facilitates the transcription of antioxidant genes, including heme oxygenase-1 (HO-1), NAD(P)H: quinone oxidoreductase 1 (NQO1), and superoxide dismutase (SOD)[18, 19]. These enzymes contribute to scavenging free radicals and subsequently attenuating inflammation. Moreover, Nrf2 has been reported to inhibit the expression of genes encoding pro-inflammatory cytokines [20]. Therefore, Nrf2 is a critical target not only for antioxidant intervention but also for the prevention and treatment of inflammation.

*Lespedeza bicolor*, a deciduous shrub from the Leguminosae family, is commonly found in temperate regions across the United States, Australia, and Asia. It has been utilized for its medicinal properties, particularly in the treatment of urinary tract inflammation, nephritis, and diabetes [21]. Moreover, various components derived from *L. bicolor* roots, including alkaloids, flavonoids, sterols, and terpenoids, have exhibited potential pharmacological activities, such as inhibition of bacterial neuraminidase, suppression of cancer cell proliferation, antioxidant properties, and anti-inflammatory effects [22-25]. In previous work, we successfully isolated a novel flavonoid derivative, 8-methoxybicolosin C (8-MC), from *L. bicolor* roots and elucidated its structure (Fig. S1). Furthermore, we confirmed the anticancer effects of 8-MC [26]. Despite these promising initial findings, the anti-inflammatory and antioxidant properties of 8-MC have remained largely unexplored. In the present study, we address this gap by investigating for the first time the specific molecular mechanisms underlying the anti-inflammatory and antioxidant activities of 8-MC in LPS-induced mouse Kupffer cells (ImKCs). Our findings uniquely demonstrate that 8-MC exerts protective effects by directly binding Keap1, stabilizing the Nrf2 protein, and thereby enhancing its nuclear translocation. Consequently, 8-MC increases the expression of antioxidant enzymes, particularly HO-1, while simultaneously suppressing the activation of the MAPK and NF-κB signaling pathways. These novel insights into the molecular action of 8-MC highlight its significant potential as a therapeutic agent capable of modulating critical pathways involved in oxidative stress and inflammation.

## Materials and Methods

### Isolation and Identification of 8-MC

The isolation of 8-MC from *L. bicolor* roots was performed as previously described [26]. Briefly, dried *L. bicolor* roots underwent ultrasonication and were extracted three times with pure methanol (MeOH). The extract was then fractionated with ethyl acetate (EtOAc) and further separated into four fractions (E1−E4) through silica gel column chromatography (CC) using a dichloromethane (DCM)–MeOH gradient. The E1 fraction was subsequently separated into six subfractions (H1−H6) using silica gel CC with a hexane–EtOAc gradient. Subfraction H2 was further purified using reversed-phase C18 medium-pressure liquid chromatography (RP C18-MPLC) with a MeOH gradient (10%-100%), resulting in five additional subfractions (M1−M5). The 8-MC was finally purified from subfraction M2 by preparative high-performance liquid chromatography (HPLC) using a mobile phase of acetonitrile (CH_3_CN) and water (H_2_O) in a 7:3 ratio. The purity of 8-MC was determined using an HPLC system (Shimadzu Corp., Japan) featuring an SPD-20A UV/V is detector and an LC-20AR solvent pump. An Atlantis T3 Column (4.6 mm × 250 mm, 5 μm, Waters Corp., USA) was used for chromatographic analysis, and signals were detected at 210 nm. Elusion was carried out at a flow rate of 1 ml/min under a linear gradient using H_2_O (A) and CH_3_CN (B) as mobile phases: 10% to 90% B from 0 to 30 min, and 90% to 100% B from 30 to 40 min. The sample injection volume was set to 5 μl. The purity of the compound in the study was determined to be 99%.

### Cell Culture

ImKCs were sourced from Sigma-Aldrich (USA). The cells were grown in Dulbecco’s modified Eagle’s medium (DMEM; WELGENE Inc., Republic of Korea) containing 10% fetal bovine serum (FBS; Gibco, USA) and 1%penicillin-streptomycin (Corning Inc., USA), maintained at 37°C in a humidified CO_2_ incubator. For this study, we used the cells at passages 2 or 3 to ensure cell stability after thawing.

### Evaluation of Cell Viability

Cell viability was evaluated under the manufacturer's protocol using the EZ-Cytox Cell Viability Assay Kit (DoGenBio, Republic of Korea). In brief, ImKCs were cultured in 96-well plates (3 × 10^4^ cells) and exposed to 8-MC (prepared by serial dilution at 1.25, 2.5, 5, 10, and 20 μM) for 24 h. The EZ-Cytox reagent was added at a ratio of 1/10 of the culture medium and incubated again in an incubator at 37°C for 20 min. Cell viability was evaluated using a plate reader (Synergy HTX; BioTek Instruments, USA) at an optical density of 450 nm.

### Evaluation of Nitric Oxide (NO) Levels

After culturing ImKCs on 12-well plates (4 × 10^5^ cells), the cells were pre-treated with 8-MC (prepared by serial dilution at 1.25, 2.5, 5, 10, and 20 μM) for 2 h, followed by additional treatment with LPS (0.5 μg/ml) for 24 h. The supernatants were collected and mixed with Griess reagents (1% sulfanilamide, 0.1% N-1-naphthylethylenediamine dihydrochloride, and 2.5% phosphoric acid) in equal proportions and allowed to react. To quantify NO production, absorbance was measured at 540 nm using a microplate reader, and the calculation method was previously described in detail (Antioxidant 2023).

### Protein Extraction and Immuno Blot Analysis

After culturing ImKCs on 12-well plates (4 × 10^5^ cells), the cells were treated with 8-MC (prepared by serial dilution at 1.25, 2.5, 5, 10, and 20 μM) for 2 h, followed by treatment with LPS (0.5 μg/ml) for 15 min (to assess MAPK and IκB levels), or 24 h (to assess iNOS, HO-1, and Nrf2 expression). Then, a radioimmunoprecipitation assay (RIPA) buffer (Biosesang, Republic of Korea) comprising a protease inhibitor cocktail (P8340) and phosphatase inhibitor cocktail (II, P5726; III, P0044; Sigma-Aldrich) was used to lyse the cells. After measuring its concentration, the protein was denatured by incubation at 100°C for 10 min. Following separation by SDS-PAGE, the denatured protein was transferred to polyvinylidene fluoride (PVDF) membranes, which were then incubated with 5% non-fat dry milk at room temperature (RT) for 1 h. After blocking, the membranes were exposed overnight at 4°C with specific primary antibodies (Table S2). After washing, the membranes were exposed to horseradish peroxidase (HRP)-labeled anti-rabbit or anti-mouse (Cell Signaling Technology) antibody for 1 h at RT. Protein expression was visualized using an enhanced chemiluminescence solution (DoGenBio) detected using the Amersham Imager 680 system (GE Healthcare, USA) and analyzed with Image Quant TL software (GE Healthcare).

### Assessment of Pro-Inflammatory Cytokine Secretion

The measurement of pro-inflammatory cytokine secretion in cell supernatants was conducted using an ELISA kit. After culturing ImKCs on 12-well plates (4 × 10^5^ cells), the cells were pre-treated with 8-MC (prepared by serial dilution at 1.25, 2.5, 5, 10, and 20 μM) for 2 h. The cells were then treated with LPS (0.5 μg/ml) for 24 h. For cytokine detection, 96-well plates were coated with purified antibodies (IL-6; BD Pharmingen, USA) (IL-1β, TNF-α; Thermo Fisher Scientific Inc., USA) and incubated overnight at 4°C. This was followed by a 1-h incubation at RT in 1% bovine serum albumin (BSA) to minimize non-specific binding. The cell culture supernatants were incubated in 96-well plates coated with antibodies for 2 h. After that, the detection antibodies were added to the plate and incubated for 1 h. Then, the plate was washed and the streptavidin-HRP solution (BD Pharmingen) was added and reacted in the dark for 30 min. Finally, TMB substrate solution was added to the wells in the dark, after which the reaction was terminated by adding a stop solution (2N H_2_SO_4_), and the absorbance at 450 nm was quantified using a microplate reader (Synergy HTX).

### Immunofluorescence Assay

After culturing ImKCs on a coverslip coated with poly-L-lysin (Sigma-Aldrich), the cells were treated with 8-MC (10 μM) for 2 h, followed by treatment with LPS (0.5 μg/mL) for 10 min. After that, the cells were fixed with 4%paraformaldehyde (Biosesang) for 10 min before being permeabilized using 0.2% Triton X-100 for 10 min. To block non-specific binding, the cells were incubated with 1% BSA for 30 min. Next, the cells were incubated with anti-NF-κB (Santa Cruz Biotechnology Inc.) or anti-Nrf2 (Cell Signaling Technology) antibodies at 4°C overnight. After three washes, the cells were labeled with Alexa-555-labeled anti-mouse or anti-rabbit (Invitrogen, USA) antibody, followed by staining with 4',6-diamidino-2-phenylindole (DAPI) to label nuclei. Finally, the cells were mounted with a mounting medium (Thermo Fisher Scientific Inc.), and fluorescence images were acquired using a Nikon AX R confocal microscope (Nikon Instruments, Japan).

### Glutathione Assay

A glutathione assay kit (DoGenBio) was employed to determine the levels of glutathione (GSH) and oxidized glutathione (GSSG). ImKCs were cultured on 12-well plates (4 × 10^5^ cells) followed by treatment with 8-MC (prepared by serial dilution at 1.25, 2.5, 5, and 10 μM) for 2 h, and stimulation with LPS (0.5 μg/ml) for 24 h. The cells were collected by centrifugation, and the cell pellet was homogenized in 1 ml of cold MPA (metaphosphoric acid) buffer. Then, the cells were centrifuged at 10,000 ×*g* for 15 min at 4°C. Cell lysates were collected and divided into two separate tubes: one for measuring GSH and the other for GSSG. A solution of 2-vinyl pyridine diluted to 5% M was added exclusively to the GSSG sample. Then, a mixture of 40% 5,5'-dithio-bis-2-nitrobenzoic, glutathione reductase (250 U/ml), and 40% NADPH was added to each sample. The absorbance was measured at 412 nm to quantify GSH and GSSG levels.

### Molecular Docking Analysis

The structure of 8-MC was generated using ChemDraw 20.0 (Revvity Inc., USA), and energy minimization was carried out using MM2 force field in Chem3D 20.0 (Revvity). The minimized structure was subsequently saved in Mol2 format. The Kelch domain of Keap1 (PDB code: 4XMB) was obtained from the RCSB Protein Data Bank (www.rcsb.org/) with a resolution of 2.43 Å. The center of the docking grid box (23.25 Å × 20.25 Å × 19.5 Å) was defined using the geometry center of the 12e ligand [2,2'-(naphthalene-1,4-diylbis(((4-methoxy phenyl)sulfonyl) azanediyl))diacetamide) [27] in the crystal structure, determined with the GetBox plugin in PyMOL (Schrödinger Inc., USA). Molecular docking of 8-MC with Keap1 was conducted using the LeDock program (www.lephar.com), treating the ligand as flexible and keeping the receptor rigid. From the thirteen generated docking poses, the optimal binding structure was selected by evaluating molecular interaction and docking affinity. The conformation with the lowest binding energy was considered the optimal binding conformation. To visualize the docking data, PyMOL and LigPlot+ software were employed (European Bioinformatics Institute, UK).

### Statistical Analysis

The Mann-Whitney U test, a non-parametric statistical analysis, was performed using SPSS Statistics 27 (IBM Corp. Inc., USA) to determine significance. Data are expressed as the mean ± SEM, derived from three independent experiments, and statistical significance was defined as *p* < 0.05. Additionally, untreated and LPS-stimulated groups were included as experimental controls, and sample sizes were determined based on statistical considerations.

## Results

### Effects of 8-MC on ImKC Viability

**Fig. 1:**
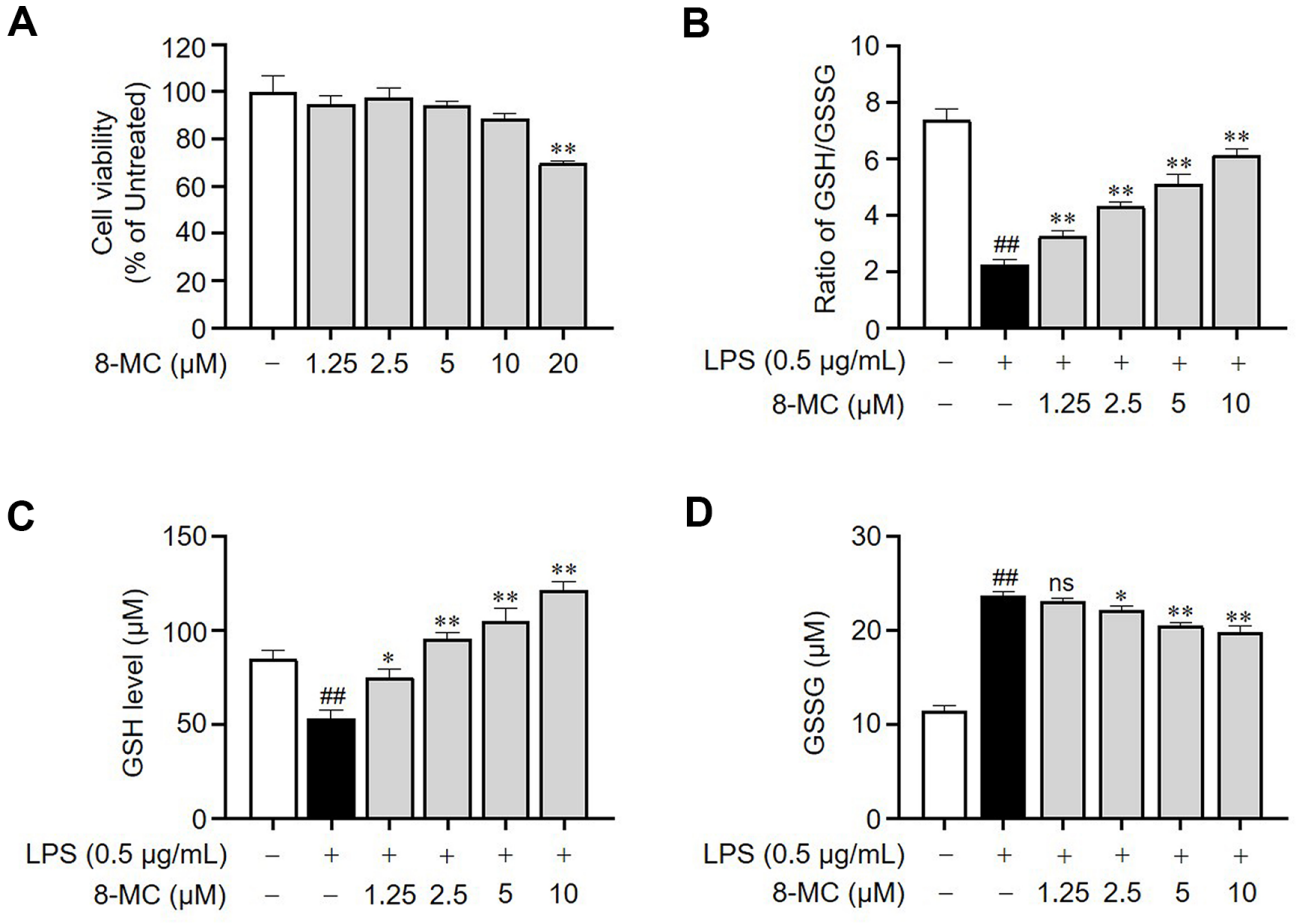
Effect of 8-MC on cell viability and GSH/GSSG ratio. ImKCs were treated with 8-MC at the indicated concentrations for 24 h. (**A**) The results of cell viability were assessed and presented as a percentage in comparison to the untreated group. The cells were pre-treated with 8-MC at the indicated concentrations for 2 h and then induced with lipopolysaccharide (LPS, 0.5 μg/ml) for 24 h. The levels of reduced glutathione (GSH) (**B**), oxidized glutathione (GSSG) (**C**), and the GSH/GSSG ratio (**D**) were calculated based on these measurements. Each result represents the average value ± SEM from three individual replicates. ##*p* < 0.01 in comparison with the untreated group. *p < 0.05, **p < 0.01 in comparison with the LPS-treated group. ns, not significant. ImKCs, immortalized Kupffer cells; 8-MC, 8-methoxybicolosin C.

In previous studies, we isolated and purified 8-MC from *L. bicolor* roots [26]. Additionally, we elucidated the structure and confirmed the purity of 8-MC (Fig. S1A and S1B). To evaluate the effect of 8-MC on cells, various concentrations of 8-MC were used to treat ImKCs for 24 h. The cell viability analysis results indicated no significant impact on cell viability when exposed to 8-MC concentrations below 10 μM (Fig. 1A). Accordingly, all subsequent experiments utilized 8-MC concentrations up to 10 μM.

### Treatment with 8-MC Increases the GSH Level in LPS-Induced ImKCs

Macrophages stimulated with LPS are well known to induce inflammation and oxidative stress [28]. In this regard, we investigated whether 8-MC could exert antioxidant and anti-inflammatory effects in LPS-induced ImKCs. To evaluate the antioxidant effects of 8-MC, our focus was on assessing its impact on the ratio of GSH to GSSG in LPS-induced ImKCs. GSH is vital for maintaining physiological ROS levels and plays a key role in the cellular defense mechanism against oxidative stress [29]. The findings revealed a dose-dependent elevation in the GSH/GSSG ratio following treatment with 8-MC (Fig. 1B). Specifically, exposure of ImKCs to LPS alone led to a reduction in GSH levels and an accumulation of GSSG due to oxidative stress compared to the control condition. However, treatment with 8-MC successfully countered the LPS-induced reduction in GSH levels in a concentration-dependent manner while also promoting GSH production (Fig. 1C and 1D). These findings indicate that 8-MC may possess significant antioxidant properties.

### 8-MC acts to Inhibit Both iNOS Expression and NO Generation in LPS-Induced ImKCs

**Fig. 2:**
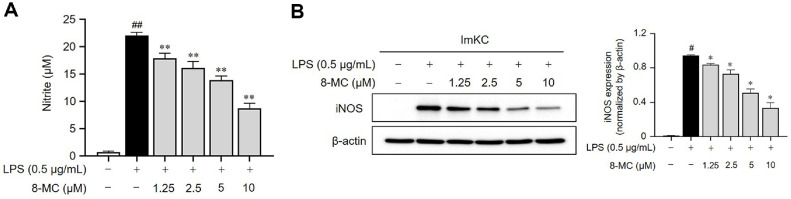
The impact of 8-MC on iNOS-driven nitric oxide (NO) production in LPS-induced ImKCs. ImKCs were pre-treated with 8-MC at the indicated concentrations for 2 h and then induced with LPS (0.5 μg/ml) for 24 h. (**A**) The levels of NO production were assessed by utilizing Griess reagents. (**B**) Protein expression levels were assessed via western blot analysis (WB) and normalized using β-actin. Each result represents the average value ± SEM from three individual replicates. #*p* < 0.05, ##*p* < 0.01 in comparison with the untreated group. *p < 0.05, *p < 0.01 in comparison with the LPS-treated group.

To evaluate the anti-inflammatory potential of 8-MC, we analyzed NO production in LPS-induced ImKCs. NO is fundamental to the signaling events that orchestrate the onset and progression of inflammation [30]. ImKCs were exposed to varying concentrations of 8-MC with the results revealing that LPS stimulation significantly increased NO production compared to the untreated group. However, treatment with 8-MC resulted in a dose-dependent decrease in NO production (Fig. 2A). Additionally, we assessed the expression of iNOS, which regulates NO synthesis [31]. Remarkably, ImKCs treated with varying concentrations of 8-MC exhibited a dose-dependent reduction in iNOS expression (Fig. 2B). Collectively, these findings indicated that 8-MC attenuates NO production by inhibiting iNOS expression, suggesting its potential anti-inflammatory effects.

### 8-MC Acts to Inhibit the LPS-Induced Release of Pro-Inflammatory Cytokines

**Fig. 3:**
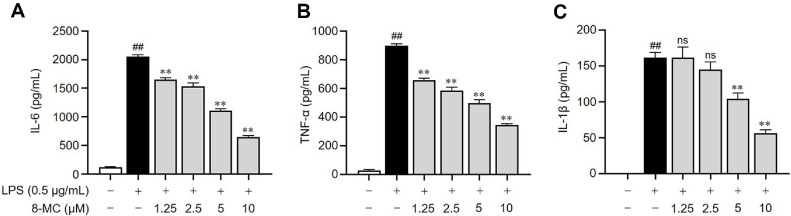
Pro-inflammatory cytokine inhibition effect of 8-MC in LPS-induced ImKCs. ImKCs were pre-treated with 8-MC at the indicated concentrations for 2 h and then induced with LPS (0.5 μg/ml) for 24 h. (A-C) The secretion of interleukin (IL)-6, tumor necrosis factor (TNF)-α, and IL-1β in the cell supernatant was assessed through ELISA analysis. Each result represents the average value ± SEM from three individual replicates. ##*p* < 0.01 in comparison with the untreated group. **p < 0.01 in comparison with the LPS-treated group. ns, not significant.

Pro-inflammatory cytokines are secreted in LPS-activated macrophages and promote the progression of inflammation [32]. To evaluate the anti-inflammatory potential of 8-MC, we measured the levels of pro-inflammatory cytokines, namely IL-6, TNF-α, and IL-1β. We observed a notable increase in the secretion of these inflammatory cytokines in LPS-induced ImKCs compared to the untreated group. Conversely, in ImKC cells exposed to 8-MC, we observed a dose-dependent reduction in the secretion of all these inflammatory cytokines (Fig. 3A-3C). These findings demonstrate the ability of 8-MC to suppress LPS-induced cytokine production and highlight its anti-inflammatory capabilities.

### 8-MC Induces the Activation of the Nrf2/HO-1 Pathway for Antioxidant and Anti-Inflammatory Effect

**Fig. 4:**
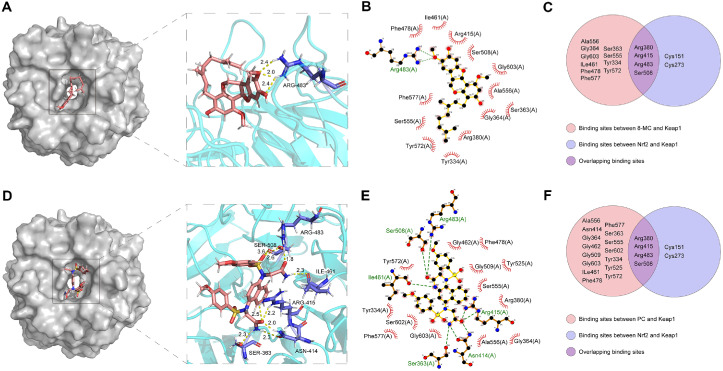
Comparative molecular docking analysis of 8-MC and PC with Keap1. (**A–C**) Binding mode of 8-MC with Keap1: (**A**) 3D binding complex showing hydrogen bonds formed at Arg483, (**B**) 2D interaction map highlighting hydrogen bonds and hydrophobic contacts, and (**C**) Venn diagram showing overlapping binding residues with Nrf2. (**D–F**) Binding mode of the positive control (PC) with Keap1: (**D**) 3D complex visualization, (**E**) detailed interaction map, and (**F**) Venn diagram displaying the overlap of binding sites between PC and Nrf2 on Keap1. These results suggest that both 8-MC and PC may competitively occupy the Nrf2-binding interface on Keap1.

We observed in our current findings that 8-MC exerts notable efficacy in mediating anti-oxidative and anti-inflammatory effects. However, the precise molecular mechanisms governing these effects remain elusive. Therefore, further investigation is warranted to comprehensively elucidate the underlying regulatory pathways. Notably, Nrf2 serves as a major transcription factor involved in the modulation of numerous genes with antioxidant and anti-inflammatory properties [33]. Under basal conditions, Nrf2 is bound to Keap1, inhibiting its translocation into the nucleus. To explore whether 8-MC directly interacts with Keap1 and disrupts its binding with Nrf2, we conducted a molecular docking analysis. The results revealed a binding affinity of -6.90 kcal/mol between 8-MC and Keap1 protein (Table S1). As shown in Fig. 4A, 8-MC fits into the binding pocket of Keap1, establishing a hydrogen bond with the Arg483 residue. Furthermore, Keap1 interacts hydrophobically with 13 amino acid residues on the 8-MC molecule, including Phe478, Ile461, Arg415, Ser508, Gly603, Ala556, Ser363, Gly364, Arg380, Tyr334, Tyr572, Ser555, and Phe577 (Fig. 4B). Previous research has identified key amino acids, such as Arg380, Arg415, Arg483, Cys151, Cys273, and Ser508 as critical in Keap1’s regulation of Nrf2 activity [34, 35]. The Venn diagram in Figure 4C provides an overview of the molecular docking interactions, highlighting the shared residues. Moreover, to verify the validation of our docking method, we redocked the co-crystallized ligand PC (2,2'-(naphthalene-1,4-diylbis(((4-methoxyphenyl)sulfonyl)azanediyl))diacetamide) into the Keap1 Kelch domain with identical docking parameters used for 8-MC. The redocking complex exhibited a binding energy of −11.82 kcal/mol, and established hydrogen bonds with Arg415, Arg483, Asn414, Ser363, Ser508, and Ile461 residues (Table S1; Fig. 4D-4F). We also observed overlap amino acid Arg483 in both docking complexes (Fig. 4B and 4E). These results demonstrated that 8-MC was bound to the site of the Keap1 Kelch domain. Overall, our molecular docking study suggests that 8-MC has the potential to competitively bind to Keap1, which may facilitate the release of Nrf2 from Keap1. However, to confirm the biological relevance of this interaction, it is essential to perform surface plasmon resonance (SPR) and co-immunoprecipitation (Co-IP) assays in future studies.

**Fig. 5:**
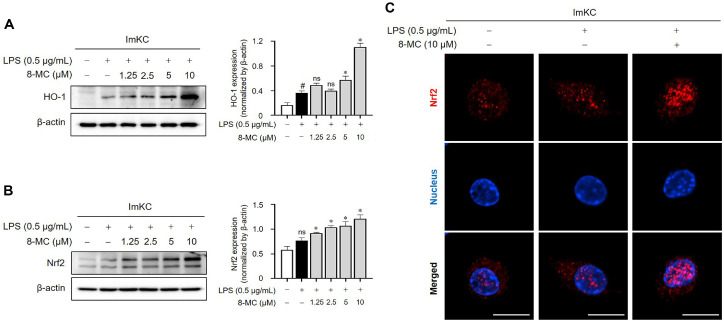
Upregulation of HO-1 and Nrf2 expression by 8-MC in LPS-induced ImKCs. ImKCs were pre-treated with 8-MC at the indicated concentrations for 2 h and then induced with LPS (0.5 μg/ml) for 4 h (B and C) or 24 h (**A**). (**A** and **B**) Protein expression levels were assessed via the WB and normalized using β- actin. Each result represents the average value ± SEM from three individual replicates. (**C**) A representative image captured via immunofluorescence depicts the movement of Nrf2 (red) into the DAPI-stained nucleus (blue). The image scale is set to 10 μm. #*p* < 0.05 in comparison with the untreated group. ns, not significant. *p < 0.05 in comparison with the LPS-treated group.

HO-1, an enzyme facilitating heme degradation, exhibits antioxidant properties due to its regulatory relationship with Nrf2 [36]. Additionally, previous research has indicated the pivotal role of HO-1 in macrophages, where it alleviates inflammatory diseases through the degradation of iNOS [37] and the regulation of NO production [36]. Consequently, we sought to determine whether the Nrf2/HO-1 signaling pathway contributes to the antioxidant and anti-inflammatory effects of 8-MC. Remarkably, treatment with 8-MC resulted in a notable increase in HO-1 protein in LPS-induced ImKCs (Fig. 5A). Moreover, 8-MC exhibited a dose-dependent upregulation of Nrf2 expression, a crucial transcription factor regulating HO-1 (Fig. 5B). Immunofluorescence analysis further confirmed that 8-MC promotes the nuclear translocation of Nrf2, as evidenced by its substantial accumulation of Nrf2 in the nucleus (Fig. 5C). These results suggest that the anti-inflammatory and antioxidant properties of 8-MC are closely associated with the activation of the Nrf2/HO-1 signaling pathway.

**Fig. 6:**
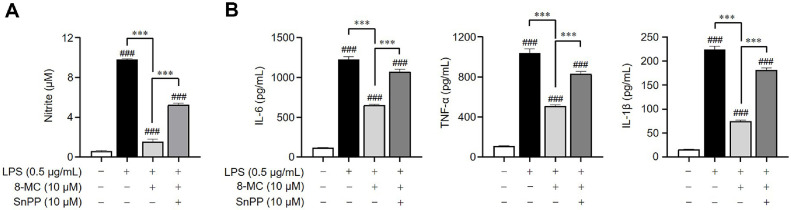
ImKCs were pre-treated with 8-MC (10 μM) for 2 h, with or without Sn protoporphyrin (SnPP, 10 μM), and then induced with LPS (0.5 μg/ml) for 24 h. (**A**) The levels of NO production were assessed by utilizing Griess reagents. (**B**) The secretion of pro-inflammatory cytokines in the cell supernatant was assessed through ELISA analysis. Each result represents the average value ± SEM from three individual replicates. ###*p* < 0.001 in comparison with the untreated group. ***p < 0.001 in comparison with the LPS-treated group.

To investigate the correlation between the anti-inflammatory effects of 8-MC and HO-1 expression, we utilized Sn protoporphyrin (SnPP), an HO-1 inhibitor, in LPS-induced ImKCs and evaluated the anti-inflammatory properties of 8-MC. When HO-1 expression was suppressed by SnPP, the ability of 8-MC to reduce LPS-induced NO production was notably weakened (Fig. 6A). Moreover, the inhibitory effects of 8-MC on pro-inflammatory cytokine secretion were also diminished in the presence of SnPP (Fig. 6B). These results demonstrate a clear connection between the anti-inflammatory effects of 8-MC and HO-1 expression. Consequently, it can be inferred that 8-MC exerts its anti-inflammatory effects largely by activating the Nrf2/HO-1 pathway.

### 8-MC Suppresses Inflammatory Responses through Regulation of the NF-κB and MAPK Pathways

**Fig. 7:**
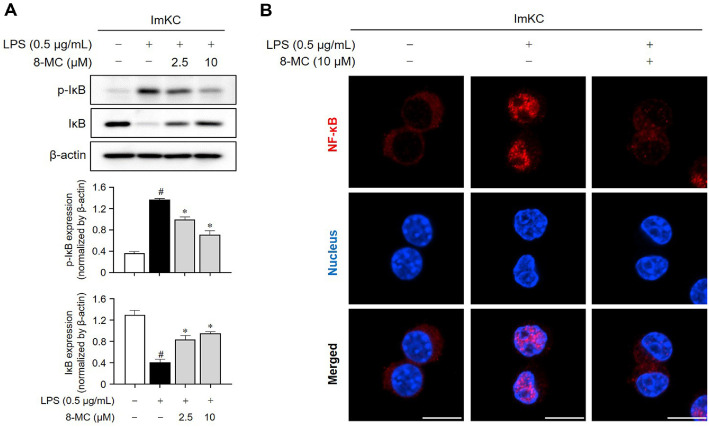
Inhibition of IκB degradation and NF-κB translocation by 8-MC in LPS-induced ImKCs. ImKCs were pre-treated with 8-MC (2.5 and 10 μM) for 2 h and then induced with LPS (0.5 μg/ml) for 10 min. (**A**) Protein expression levels were assessed via the WB and normalized using β-actin. Each result represents the average value ± SEM from three individual replicates. (**B**) A representative image captured via immunofluorescence depicts the movement of NF-κB (red) into the DAPIstained nucleus (blue). The image scale is set to 10 μm. #*p* < 0.05 in comparison with the untreated group. *p < 0.05 in comparison with the LPS-treated group.

We investigated the potential of 8-MC in inhibiting the translocation of NF-κB, a transcription factor known for its role in modulating inflammation-related expression in macrophages [38]. In our investigation we sought to elucidate the impact of 8-MC on NF-κB activity by evaluating IκB phosphorylation and NF-κB nuclear translocation in LPS-induced ImKCs. Initially, we observed a rise in IκB phosphorylation upon LPS treatment. However, administration of 8-MC suppressed this phosphorylation response in LPS-induced ImKCs (Fig. 7A). Subsequently, we conducted immunofluorescence staining to directly visualize NF-κB nuclear translocation. Notably, LPS treatment markedly facilitated NF-κB translocation into the nucleus compared to the untreated group, a phenomenon substantially mitigated by 8-MC administration in LPS-induced ImKCs (Fig. 7B). Taken together, these results strongly support the role of 8-MC in regulating inflammatory responses by suppressing NF-κB activation.

**Fig. 8:**
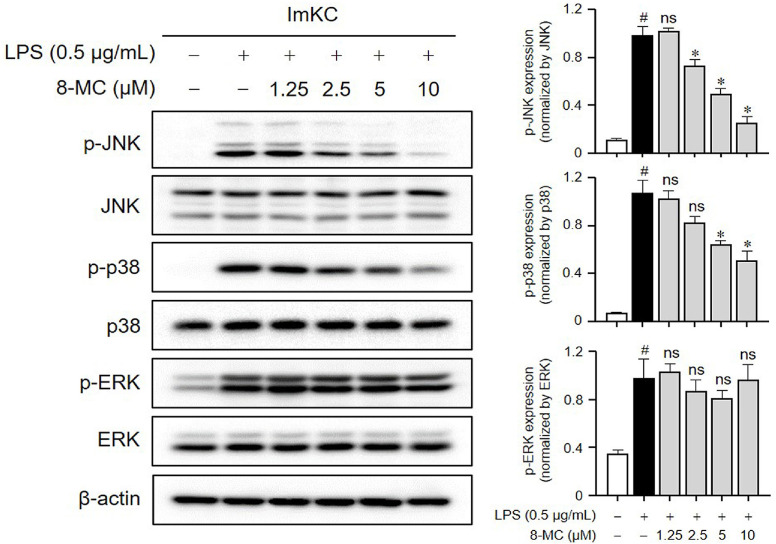
Inhibition of MAPK phosphorylation by 8-MC in LPS-induced ImKCs. ImKCs were pre-treated with 8-MC at the indicated concentrations for 2 h and then induced with LPS (0.5 μg/ml) for 10 min. Protein expression levels were assessed via the WB and normalized using β-actin. The phosphorylation levels of each protein were individually adjusted by normalizing them with the total levels of p38, JNK, and ERK. Each result represents the average value ± SEM from three individual replicates. #*p* < 0.05 in comparison with the untreated group. *p < 0.05 in comparison with the LPS-treated group. ns, not significant.

The MAPK pathways, including JNK, p38, and ERK, play critical roles in mediating inflammatory responses, typically triggered by LPS-induced phosphorylation [39]. To elucidate the underlying mechanism responsible for the anti-inflammatory effects of 8-MC, we investigated the activation of the MAPK pathway in LPS-induced ImKCs. Following LPS treatment, there was a significant elevation in the phosphorylation levels of JNK, p38, and ERK compared to the control group. Interestingly, 8-MC exhibited a specific and dose-dependent inhibition of the p38 and JNK pathways (Fig. 8). These results showed that the anti-inflammatory effect of 8-MC correlates with the inhibition of p38 and JNK in the MAPK pathway.

## Discussion

The liver is a vital organ responsible for detoxification, metabolism, bile production, and immune regulation, playing a central role in maintaining systemic homeostasis [40, 41]. Kupffer cells, the resident macrophages of the liver, serve as sentinels of hepatic immunity by regulating both protective and inflammatory responses [42, 43]. However, during chronic liver injury, excessive activation of Kupffer cells contributes to sustained inflammation and oxidative stress, which are key drivers of liver pathologies such as fibrosis, non-alcoholic steatohepatitis (NASH), and ultimately cirrhosis [44, 45]. Moreover, infiltrating monocyte-derived macrophages have also been reported to play a crucial role in liver pathology by promoting extracellular matrix deposition and chronic inflammation [46]. In this study, we investigated the mechanistic basis of the antioxidant and anti-inflammatory effects of 8-MC in LPS-stimulated immortalized mouse Kupffer cells (ImKCs), which serve as a model for resident Kupffer cells.

In recent studies, researchers have actively sought potential therapeutic candidates to prevent liver diseases caused by inflammation and oxidative stress [47-49], and interest in this field continues to grow steadily. In this study, we employed the naturally occurring compound 8-MC, which is characterized by a pterocarpan structure and has been reported to be commonly found in the *L. bicolor* family [22, 25]. In addition, 8-MC belongs to the flavonoid family—secondary metabolites predominantly synthesized by plants that contain diverse phenolic structures [50]—and is well known for its antioxidant, anti-inflammatory, and anticancer properties [51]. Nevertheless, our understanding of the complex mechanisms underlying flavonoid actions remains incomplete. Recent studies have reinforced the therapeutic relevance of flavonoids by demonstrating their ability to modulate key redox and inflammatory pathways, particularly Nrf2/HO-1 signaling, in various disease models. For instance, flavonoids have been shown to alleviate oxidative stress and inflammation in metabolic, cardiovascular, pulmonary, and age-related inflammatory disorders through activation of the Nrf2 axis [52-56]. These findings collectively emphasize the central role of Nrf2/HO-1 modulation in flavonoid-mediated protection across tissues. Building upon this evidence, our study is the first to present novel insights into the antioxidant and anti-inflammatory effects of 8-MC, as well as its underlying mechanisms, in LPS-stimulated ImKCs.

Our study demonstrated that 8-MC increased the GSH/GSSG ratio in LPS-treated ImKCs, thereby improving the cellular redox balance (Fig. 1). Additionally, 8-MC effectively suppressed the production of nitric oxide (NO), a key inflammatory mediator, as well as pro-inflammatory cytokines (Figs. 2 and 3). Furthermore, 8-MC was found to promote the expression of heme oxygenase-1 (HO-1), a critical component of the cellular defense system [57], along with the expression and nuclear translocation of its regulatory transcription factor Nrf2 (Fig. 5)[58, 59]. These findings suggest that 8-MC possesses dual properties capable of alleviating both oxidative stress and inflammation. Notably, the inhibition of HO-1 activity reduced the anti-inflammatory effects of 8-MC (Fig. 6), indicating that its cytoprotective effects are dependent on the activation of the Nrf2/HO-1 pathway. In particular, our molecular docking assay provides novel evidence that 8-MC directly binds to Keap1, the binding site for Nrf2 (Fig. 4). This direct interaction strongly supports the hypothesis that 8-MC exerts it cytoprotective effects by binding to Keap1 to prevent Nrf2 degradation, thereby enabling a more direct activation of Nrf2 compared with other know Nrf2 inducers. Because Nrf2 activation is widely recognized as a key mechanism for enhancing cellular defense against oxidative stress and inflammation [58, 59], and pharmacological Nrf2 activators can alleviate liver inflammation and fibrosis [60, 61]. These results collectively indicate that 8-MC mitigates LPS-induced oxidative stress and inflammation by modulating the Nrf2/HO-1 pathway. Consequently, 8-MC holds potential as a therapeutic Nrf2 activator for managing inflammatory liver diseases. However, further studies are needed to clarify how the 8-MC-Keap1 interaction precisely influences Nrf2 activation.

In this study, the intervention of HO-1 on the anti-inflammatory effect of 8-MC was proved by treating ImKCs with SnPP, an HO-1 inhibitor (Fig. 6). HO-1 is a well-known enzyme which is transcriptionally regulated by Nrf2 and involved in the regulation of LPS-induced proinflammatory responses. However, other antioxidant enzymes, including NQO1 and SOD, are also under transcriptional regulation of Nrf2 and have roles in regulating proinflammatory responses. NQO1 was reported to selectively suppress TLR ligand-induced IL-6 and IL-12 production in peritoneal macrophages [62]. The regulatory role of NQO1 for the alleviation of acute pancreatitis by reducing serum IL-1β was also reported [63]. Furthermore, the SOD3-mediated, anti-inflammatory role in skin inflammation by inhibiting the expression of proinflammatory cytokines, including TNF-α, IL-1β, IL-6, and IL-8, and SOD1-mediated suppression of proinflammatory immune responses against oxidative stress in colitis, were also reported [64, 65]. These findings suggest the possibility that 8-MC-mediated anti-inflammatory effects could be under the regulation of other antioxidant enzymes, NQO1, and SODs. Therefore, to fully understand the 8-MC-mediated antioxidant and anti-inflammatory effects in LPS-treated ImKCs, further evaluation is required for measuring the intervention of NQO1 and SOD on the 8-MC-mediated anti-inflammatory effects.

Additionally, our findings show that 8-MC suppresses the activation of LPS-induced NF-κB and MAPK signaling pathways (Figs. 7 and 8). Specifically, 8-MC exerts these effects by blocking the degradation of IκB (Fig. 7) and selectively inhibiting the phosphorylation of JNK and p38 (Fig. 8). NF-κB is a key regulator of inflammatory responses, and its excessive activation is closely associated with various liver diseases, including hepatitis and fibrosis [66]. Similarly, the MAPK signaling pathway plays a crucial role in inflammatory signal transduction, with the activation of JNK and p38 being particularly linked to hepatocyte apoptosis and fibrosis development [67, 68]. Our results are consistent with previous reports indicating that flavonoids exert anti-inflammatory effects by targeting these pathways [69-71]. However, unlike other flavonoids that broadly inhibit MAPK activation, 8-MC selectively suppressed the phosphorylation of JNK and p38 without significantly affecting the ERK signaling pathway. This selective inhibition pattern has also been observed in other flavonoids with pterocarpan-related or isoflavonoid scaffolds, such as glabridin and daphnegiravone D, which preferentially target JNK and p38 pathways without significantly affecting ERK [72, 73]. Additionally, flavonoids like apigenin and luteolin, despite structural differences from 8-MC, similarly inhibit JNK activation without affecting ERK or p38, thus contributing to their anti-inflammatory effects [74]. Structural features, including methoxy, prenyl, and hydroxyl substitutions have been suggested to influence MAPK pathway selectivity by altering kinase binding affinity or access to upstream regulatory elements [75-78]. Therefore, the selective inhibition of 8-MC likely arises from its unique pterocarpan skeleton and specific functional groups, indicating a distinct mode of MAPK regulation compared to other flavonoids. Further studies focusing on direct molecular targets are required to elucidate the mechanisms underlying this selectivity.

We confirmed that 8-MC exhibited significant antioxidant and anti-inflammatory effects in resident Kupffer cells. However, it is important to note that the entire hepatic macrophage network, including monocyte-derived macrophages, also plays a critical role in liver pathology. Previous studies have shown that recruited monocyte-derived macrophages promote extracellular matrix deposition and sustain chronic inflammation, leading to liver fibrosis [46, 79]. In contrast, Kupffer cells can adopt anti-inflammatory or reparative phenotypes in response to microenvironmental signals [80]. Although we did not directly evaluate the effects of 8-MC on monocyte-derived macrophages in this study, our findings suggest that targeting Kupffer cells alone may effectively alleviate oxidative stress-induced inflammatory responses in the liver. From a clinical perspective, selectively modulating Kupffer cells could help prevent acute inflammation from advancing to fibrosis without excessively suppressing systemic immunity. Furthermore, a Kupffer cell-targeted therapeutic strategy has the potential to act as a precise intervention that complements existing liver disease treatments while preserving peripheral immune function. Nonetheless, because monocyte-derived macrophages recruited during liver pathogenesis also play pivotal roles, further research is warranted to determine whether 8-MC exerts similar effects on these cells and induces a switch toward a reparative macrophage phenotype.

## Conclusion

This study provides novel insights into the hepatoprotective effects of 8-MC, and demonstrate its efficacy in alleviating LPS-induced inflammation and oxidative stress in resident Kupffer cells. Notably, molecular docking analysis revealed that 8-MC directly binds to Keap1, thereby disrupting the Keap1–Nrf2 interaction and providing a mechanistic basis for Nrf2/HO-1 pathway activation. Moreover, the regulatory effects of 8-MC on NF-κB and MAPK signaling further underscore its therapeutic potential in reducing inflammation and oxidative stress. These findings highlight the promise of 8-MC in treating inflammatory liver diseases, and warrant further investigation through more complex in vivo models that include both resident and infiltrating macrophages to fully elucidate the therapeutic efficacy and mechanisms of action.

## Supplemental Materials

Supplementary data for this paper are available on-line only at http://jmb.or.kr.
