# Supplementary material for: 8-Methoxybicolosin C from Lespedeza bicolor Attenuates Inflammation and Oxidative Stress via Nrf2/HO-1 and NF-κB/MAPK Pathways in Lipopolysaccharide-Induced Mouse Kupffer Cells
Source: J Microbiol Biotechnol. 2025 Aug 18;35:e2503013. doi: 10.4014/jmb.2503.03013 (PMC12375545; doi:10.4014/jmb.2503.03013)
Supplement: Supplementary file 1 [file jmb-35-e2503013-supple.pdf]

J. Microbiol. Biotechnol. 2025. 35: e2503013

https://doi.org/10.4014/jmb.2503.03013

Supplementary Figures and Tables

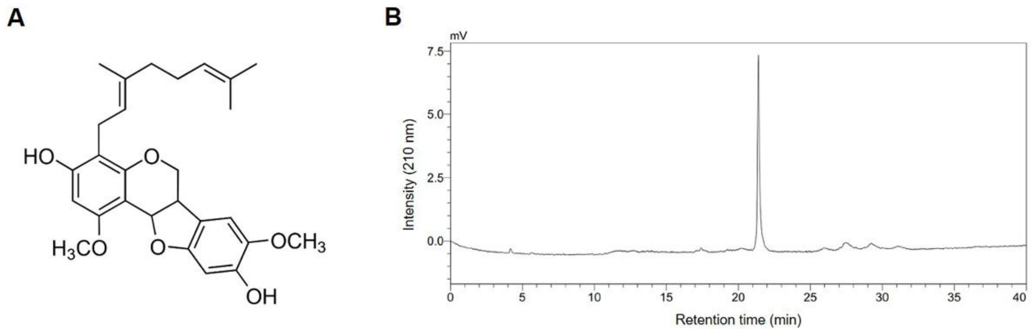

Fig. S1. Structural representation and purity evaluation of 8-methoxybicolosin (8-MC). **(A)** Illustration of the molecular structure of 8-MC. **(B)** HPLC chromatogram confirming the high purity of 8-MC.

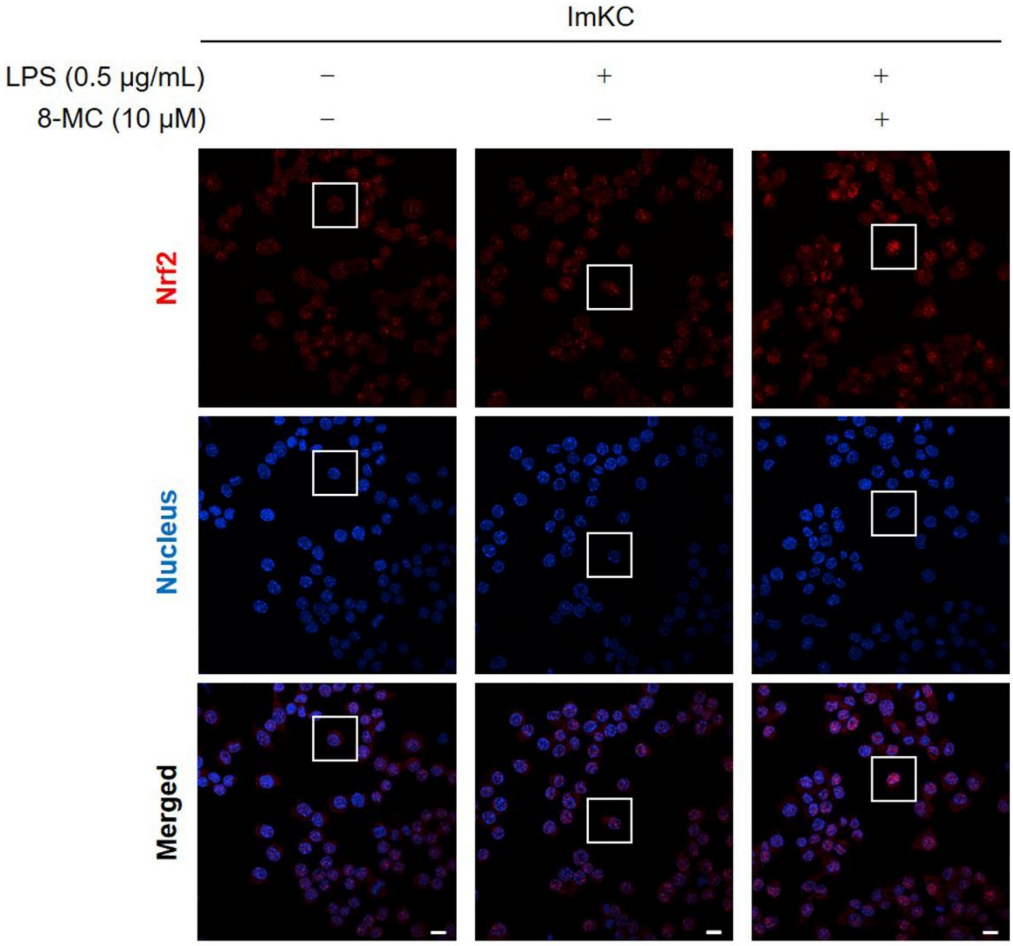

Fig. S2. Immunofluorescence visualization of Nrf2 nuclear translocation. ImKCs were treated with 8-MC (10 μM) for 2 h and then stimulated with LPS (0.5 μg/mL) for 4 h. The representative image illustrates Nrf2 (red) migration into the nucleus (blue), with nuclei counterstained using 4′,6-diamidino-2-phenylindole (DAPI). Scale bar = 10 μm.

Table S1. Molecular docking binding energy results of 8-MC and PC with Keap1 protein.

Compound	Binding energy (Kcal/mol)	Hydrogen bond Interactions
8-MC	-6.90	Arg483
PC	-11.82	Arg415, Arg483, Asn414, Ser363, Ser508, Ile461

Abbreviation: 8-MC, 8-methoxybicolosin C; PC, [2,2'-(naphthalene-1,4-diylbis(((4-methoxyphenyl)sulfonyl)azanediyl))diacetamide], the co-crystallized ligand present in the crystal structure of Keap1 (PDB ID: 4XMB).

Table S2. Information of Primary Antibodies Used for Western Blot Analysis.

Name	Company	Catalog Number
iNOS	BD Biosciences	610332
Nrf2	Cell Signaling Technology	#12721
HO-1	Santa Cruz Biotechnology Inc.	sc-390991
SAPK/JNK	Cell Signaling Technology	#9252
Phospho-SAPK/JNK	Cell Signaling Technology	#9251
p38	Santa Cruz Biotechnology Inc.	sc-7972
Phospho-p38	Cell Signaling Technology	#9211
ERK	Santa Cruz Biotechnology Inc.	sc-514302
Phospho-ERK	Cell Signaling Technology	#9101
Phospho-IκBα	Cell Signaling Technology	#9246
IkBα	Cell Signaling Technology	#4814
β-actin	Santa Cruz Biotechnology Inc.	sc-47778

Abbreviation: iNOS, inducible nitric oxide synthase; Nrf2, nuclear factor erythroid 2-related factor 2; HO-1, heme oxygenase-1; SAPK/JNK, stress-activated protein kinase/c-Jun N-terminal kinase; ERK, extracellular signal-regulated kinase; IκB, inhibitor κB.
